# Virtual Reality Aggression Prevention Therapy (VRAPT) versus Waiting List Control for Forensic Psychiatric Inpatients: A Multicenter Randomized Controlled Trial

**DOI:** 10.3390/jcm9072258

**Published:** 2020-07-16

**Authors:** Stéphanie Klein Tuente, Stefan Bogaerts, Erik Bulten, Marije Keulen-de Vos, Maarten Vos, Hein Bokern, Sarah van IJzendoorn, Chris N. W. Geraets, Wim Veling

**Affiliations:** 1 Department of Psychiatry, University Medical Center Groningen, University of Groningen, Hanzeplein 1, 9713 GZ Groningen, The Netherlands; m.vos01@umcg.nl (M.V.); c.n.w.geraets@umcg.nl (C.N.W.G.); w.veling@umcg.nl (W.V.); 2 Forensic Psychiatric Center (FPC) Dr. S. van Mesdag, Helperlinie 2, 9722 AZ Groningen, The Netherlands; heinbokern@hetnet.nl; 3 Department of Developmental Psychology, Tilburg University, Prof Cobbenhagenlaan 225, P.O. Box 90153, 5000 LE Tilburg, The Netherlands; s.bogaerts@uvt.nl; 4 Fivoor, Fivoor Science & Treatment Innovation, Kijvelandsekade 1, 3172 AB Poortugaal, The Netherlands; Sarah.van.IJzendoorn@fivoor.nl; 5 Division Diagnostics Research and Education, Forensic Psychiatric Hospital Pompefoundation, Weg door Jonkerbos 55, 6532 CN Nijmegen, The Netherlands; e.bulten@pompestichting.nl; 6 Behavioral Science Institute (BSI) of Radboud University, P.O. Box 9104, 6500 HE Nijmegen, The Netherlands; 7 Forensic Psychiatric Center (FPC) de Rooyse Wissel, P.O. Box 433, 5800AK Venray, The Netherlands; MKeulen-deVos@derooysewissel.nl

**Keywords:** aggressive behavior, forensic psychiatry, social information processing model, randomized controlled trial, virtual reality, severe psychopathology

## Abstract

Many forensic psychiatric inpatients have difficulties regulating aggressive behavior. Evidence of effective aggression treatments is limited. We designed and investigated the effectiveness of a transdiagnostic application of a virtual reality aggression prevention training (VRAPT). In this randomized controlled trial at four Dutch forensic psychiatric centers, 128 inpatients with aggressive behavior were randomly assigned to VRAPT (*N* = 64) or waiting list control group (*N* = 64). VRAPT consisted of 16 one-hour individual treatment sessions twice a week. Assessments were done at baseline, post-treatment and at 3-month follow-up. Primary outcome measures were aggressive behavior observed by staff and self-reported aggressive behavior. Analysis was by intention to treat. This trial was registered in the Dutch Trial Register (NTR, TC = 6340). Participants were included between 1 March 2017, and 31 December 2018. Compared to waiting list, VRAPT did not significantly decrease in self-reported or observed aggressive behavior (primary outcomes). Hostility, anger control, and non-planning impulsiveness improved significantly in the VRAPT group compared to the control group at post-treatment. Improvements were not maintained at 3-month follow-up. Results suggest that VRAPT does not decrease aggressive behavior in forensic inpatients. However, there are indications that VRAPT temporarily influences anger control skills, impulsivity and hostility.

## 1. Introduction

Aggression of forensic psychiatric inpatients is highly prevalent, with recent estimates between 31% and 59% of at least one violent assault during hospitalization [1,2]. Inpatient aggression threatens safety and wellbeing of both patients and staff [3,4]. Aggression regulation is a main treatment goal in forensic psychiatry, as it is a prerequisite for successful release and re-socialization. Effective aggression interventions are highly needed, but the body of research on psychosocial interventions in forensic populations is small.

A systematic review of psychological therapies designed for violent behavior in clinical and forensic settings found ten studies, providing tentative support for the effectiveness on aggressive behavior. However, the review included only two randomized controlled trials, both of cognitive behavioral therapy, one of which had negative outcomes [5]. Another more recent systematic review included 16 studies on the effect of Aggression Replacement Training (ART) on antisocial behavior in young people and adults [6]. Although results indicated positive effects of ART on recidivism rates and secondary outcomes (e.g., social skills), only four studies involved adult samples, and the majority of the included studies were of average quality because of non-randomized designs, selection bias, and small sample sizes.

The paucity of evidence on the effectiveness of aggression therapies, may be related to the characteristics of forensic populations. A meta-analysis on the efficacy of psychological treatments for violent offenders found only significant treatment effects on community recidivism, and not on institutional misconduct [7]. The authors offer several explanations for these findings, e.g., the smaller number of studies conducted in inpatient forensic mental settings. Moreover, aggression treatment is often hampered because patients have severe chronic psychiatric and aggression problems. Engaging forensic patients in treatment is challenging. Attrition is high, patients are often demoralized or unwilling to change their behavior, and have difficulties to apply therapeutic insights in their daily lives. Furthermore, previous research has shown that role-play contributed significantly to reductions in violent recidivism [7], but within forensic psychiatric settings, therapists often feel insecure provoking patients in a real-life setting.

Virtual Reality (VR) may solve several of the problems listed above. In a virtual environment, patients have the opportunity to explore their behavioral reactions to social situations and practice new behavior in a safe, controlled, realistic and personalized environment. Furthermore, VR interventions also focus on practicing behavior and not only on gaining cognitive therapeutic insights. Lastly, it is expected that VR is more enjoyable than current psychosocial therapies, which may result in better engagement in treatment, less drop-out and no-shows. We developed and tested a virtual reality aggression prevention therapy (VRAPT) targeting aggression in a sample of forensic psychiatric inpatients who stay in a highly secured setting [8].

The current study is a multicenter randomized controlled trial conducted in four forensic psychiatric centers. We investigated the effectiveness of VRAPT on aggressive behavior, by comparing VRAPT (in addition to treatment as usual) to waiting list control (treatment as usual). We hypothesized that VRAPT:

1. decreases both self-reported and staff-reported aggressive behavior;
2. reduces determinants of aggressive behavior, including anger, impulsivity, and hostility.

## 2. Methods

### 2.1. Study Design and Participants

This study was a randomized controlled trial in four forensic psychiatric centers (FPCs) in The Netherlands. Patients residing in FPCs are admitted under the judicial measure ‘TBS-order’ (in Dutch: ter-beschikking-stelling: this translates as ‘detained under hospital order’). ‘Detained under hospital order’ means that the court has established a relation between the offense committed and a psychiatric disorder. Details of the study protocol have been published elsewhere [8].

Inclusion criteria were being a forensic psychiatric inpatient, and being referred by their clinical team to the study based on pre-admission history of aggression and/or current clinical problems with reactive aggression. Exclusion criteria were (history of) epilepsy, insufficient mastery of the Dutch language, and an intelligence quotient (IQ) below 70 as estimated by an IQ test or their treating specialist.

This study has been approved by the medical ethical committee of the University Medical Centre Groningen, Groningen (number: NL52939.042.15) and was conducted according to the Declaration of Helsinki. The study was registered in the Dutch Trial Register (NTR, TC = 6340). Patients were informed about the study by a research assistant under the supervision of the head of treatment. Written informed consent was obtained, and patients were allowed to stop participating at any time without giving a reason, and without consequences for their further stay in the FPC. Participants from both groups received 35 euros for their participation on the assessments.

### 2.2. Randomization and Masking

Participants were randomly assigned to VRAPT or waiting list. An independent research coordinator of the University Medical Center Groningen performed the randomization by utilizing the program Research Randomizer. Randomization blocks of two (1:1) were generated for each participating FPC separately. Participants were informed of their allocation after the baseline measurement was completed. All assessments were done by research assistants. We aimed for assessors blinded for study condition, but this was not feasible, because all staff members and research assistants had access to clinical records, due to safety reasons and knowing the whereabouts of their patients. Furthermore, most participating patients were not allowed to leave the treatment ward without staff, so they were aware when a patient entered the VR-room. Because of this, only 30 measurements at post-measurements, and 28 measurements at follow-up were completely single-blind.

### 2.3. Procedures

**Figure 1:**
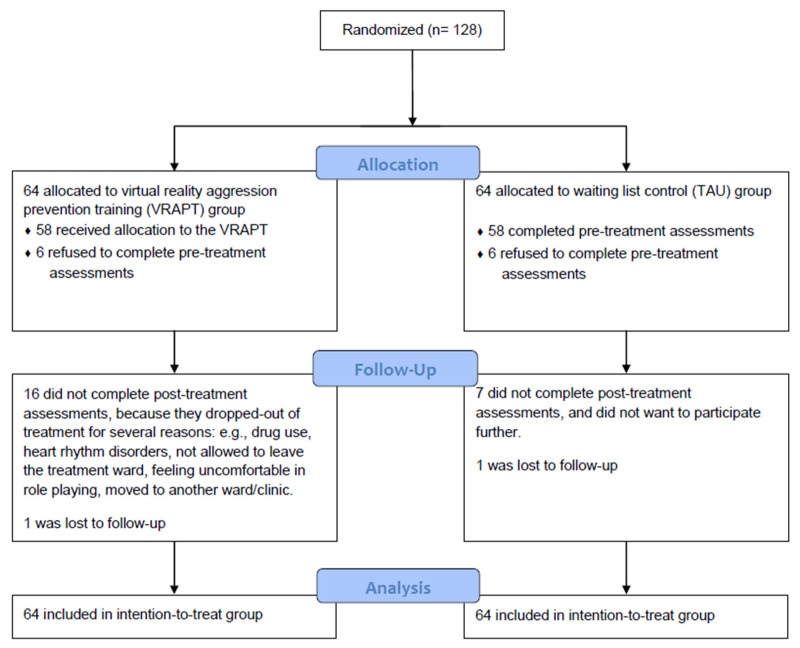
CONSORT flow diagram Virtual Reality Aggression Prevention Training (VRAPT).

Participants completed assessments at baseline (T1; pre-treatment), post-treatment (T2), and 3-month follow-up (T3). During the study period, staff was asked to complete an observation scale for each participant once weekly, to monitor aggressive behavior at the ward. Observation started three months before the intervention and lasted until end of follow-up. Participants who dropped out of the treatment were still included in the staff observation ratings. Treatment as usual consisted of standard treatments, including for instance: medication, psychomotor therapy, schema focused therapy, supportive counseling, skills training, or treatment for substance use disorders. Patients were allowed to participate as long as it did not directly target their aggression problems. Participants in the waiting list were offered VRAPT after they had completed follow-up measurements. The participants from the treatment group, completed a short interview with a research assistant at T3. See Figure 1 for the CONSORT flow diagram of VRAPT.

### 2.4. Intervention

**Figure 2:**
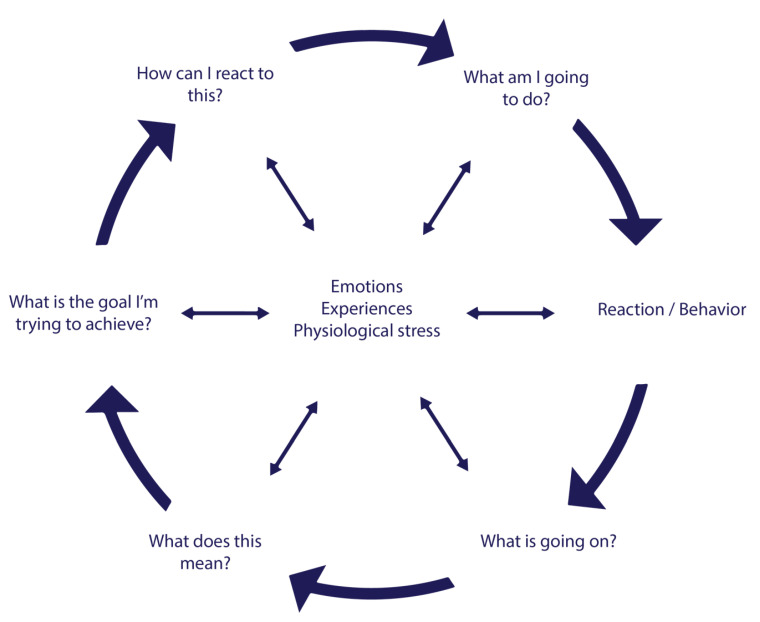
Theoretical framework of VRAPT. Based on the social information processing (SIP) model of Crick and Dodge (1994).

VRAPT consisted of 16 individual biweekly sessions that lasted on average one hour. Sessions consisted of VR exercises to practice new and adequate behavior. As a theoretical framework for VRAPT the Social Information Processing (SIP) was used. The SIP model describes mechanisms how individuals interpret and respond to social situations [9], and how these can lead to aggressive behavior. The model includes six cognitive-emotional steps, which were targeted in VRAPT (Figure 2). Except for the first introduction session, every session followed the same format: a short review of the previous session, clarification of a step of the SIP model, VR exercise, and discussing the VR exercise. Each session ended with an evaluation of the progress in learning goals [8]. Some examples of learning goals were: “I would like to learn how to react assertively, instead of aggressively. Therefore, I want to increase my skills, and to make sure I become less frustrated”, “I would learn how to regulate my anger, and react in a more prosocial manner”, “I want to discover which triggers and situations make me angry and react aggressively, so I can prevent future aggressive outbursts”. The VRAPT exercises corresponded to the different steps of the model and became more difficult as the VRAPT progressed.

The first two steps of the model are referred to as early information processing, and they are about encoding and making attributions of internal and external social cues. VR sessions 1–5 consisted of exercises on facial emotion recognition, and recognizing aggressive behavior of other people. The next four steps of the SIP model are labeled late information processing, starting with step three: goal selection. This step involves deciding the desired outcome in a given social situation. Steps four, five and six are respectively generating, evaluating and enacting responses. Recent evidence suggests that reactive aggression is associated with both early and late information processing, and deficits in information processing are related to aggressive behavior [10]. From session 6–8 VR exercises focused on de-escalating aggressive behavior of others (i.e., avatars) and on regulating physical arousal (i.e., heart rate and skin conductance). In the second half of the therapy (VR session 9–16) all SIP steps were integrated into challenging interactive virtual role-plays. The interactive virtual social scenarios were designed in an iterative process with clinicians, VR experts, software engineers and researchers. The main focus of all sessions was teaching participants to cope with provocative behavior of others more adequately and to prevent own aggressive outbursts.

**Figure 3:**
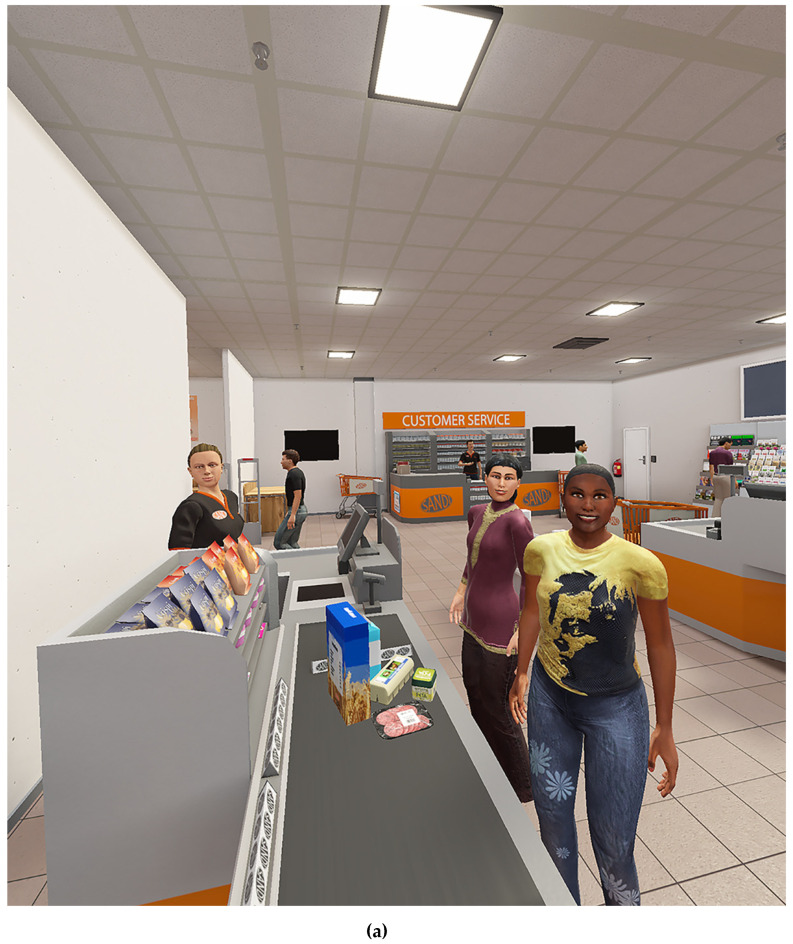

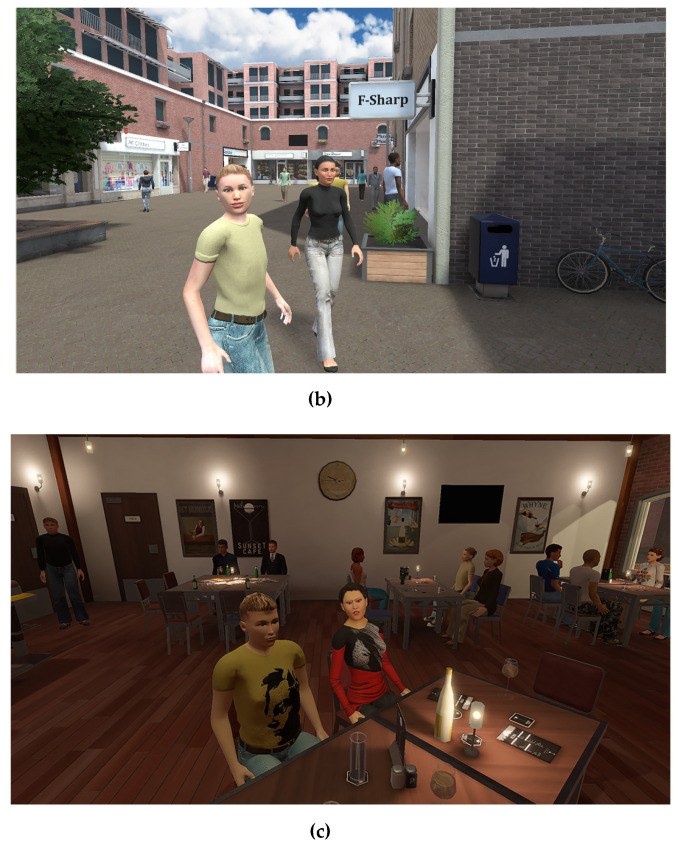
Virtual environments: supermarket (a), shopping street (b), and café (c). Source: CleVR BV, Delft, The Netherlandd.

Three virtual environments (Figure 3) were created with Unity software by CleVR BV (Delft, The Netherlands). Within the virtual environments, participants could walk around with a Microsoft Xbox One controller. During sessions, participants wore an Oculus Rift 2 (Oculus VR, California, U.S.) a head-mounted display and headphones while they were interacting with avatars. Virtual environments and avatars were controlled by the VRAPT therapist (e.g., avatars’ body movements and facial expressions). Furthermore, therapists could role-play through avatars by using a microphone with voice morphing. Because of this dynamic interactive nature of the VR software, VRAPT could be tailored to the specific needs of the participants. This allowed them to design their own learning goals and practice with specific triggers. During session 6–15, real-time heart rate (HR) and galvanic skin response (GSR) were measured and real-time displayed at the therapist interface for feedback on physical arousal. At all times, the VRAPT therapist was in control of the virtual environment and was able to immediately change and/or stop the virtual environment if necessary (e.g., in case a participant became nauseous).

VRAPT therapists were licensed psychologists and non-verbal therapists (e.g., creative therapists, psychomotor therapists). Therapists received 16 hours of training in working with the protocol and software of VRAPT from a licensed psychologist and the main author of the treatment protocol (author SKT). Therapists enrolled in VRAPT were required to have improvisations skills, and had to be familiar with role-plays. The dynamic VRAPT software allowed for personalized role-plays and exposure exercises with patients. During the training, therapists were taught how to select and play relevant social interactions, and to bring up core meanings of the aggressive situation. Furthermore, they were trained in modulating the verbal and non-verbal response of avatars in order to increase or decrease patients’ aggression and emotional responses. An advantage, according to the therapist, is that they were able to test the boundaries of aggressive behavior, without disturbing the treatment relationship. Although patients were aware that the avatar was played by the therapist, they said afterwards that they were angry at the avatar, and not at the therapist. To ensure treatment integrity, a semi-structured protocol was designed as guidance for the VRAPT sessions. In addition, monthly one-hour group videoconferences were organized for VRAPT therapists to encourage treatment fidelity and discuss ongoing treatments. Furthermore, during the interventions, therapists had to complete session forms, which were checked by the research assistants.

### 2.5. Outcomes

#### 2.5.1. Primary Outcome—Aggression

The primary outcome was level of aggressive behavior post-treatment, assessed both with staff observation and self-report. Both methods were used because they can complement each other, as a limitation of self-report questionnaires in this population is social desirability, and staff observations have limitations as aggressive behavior can occur in unobserved situations or observations can be biased. Therefore, both observation and self-report were used to allow a complete picture of the nature, degree and severity of aggressive behavior.

##### Social Dysfunction and Aggression Scale (SDAS)—Completed by Staff Members

Staff completed the 9-item Social Dysfunction and Aggression Scale (SDAS) [11,12]; weekly, from three months prior to baseline continuously until the end of follow-up. Items were scored on a 4-point scale ranging from absent (1) to severe (4). For each item, a general and peak score was scored [13]. The peak score refers to the most severe aggressive behavior, and the general score to the second most severe aggressive behavior in the same week. The SDAS peak score post-treatment (T2) was the primary outcome of the study. Research assistants collected SDAS forms on all wards on a weekly basis. In case of a missing form, they reminded the staff members. In case forms were not returned after two weeks, research assistants completed the SDAS form retrospectively, based on clinical patient files. In total, 91.5% of 4565 SDAS forms were completed timely by staff members.

##### Aggression Questionnaire (AVL)—Completed by Participants

Participants completed the Dutch version of the Aggression Questionnaire (AVL) [14]. This 29-item questionnaire assesses four sub traits of aggression, i.e., physical aggression, verbal aggression, anger and hostility [15]. The AVL total score post-treatment (T2) was the other primary outcome of the study.

#### 2.5.2. Secondary Outcomes

Secondary outcomes were used to measure constructs related to the SIP model of aggression: impulsivity, measured with the Barratt Impulsiveness Scale (BIS-11) [16]; hostility, measured with the Buss-Durkee Hostility Inventory-Dutch (BDHI-D) [17]; anger, measured with the Novaco Anger Scale and Provocation Inventory (NAS-PI) [18]; and the State-Trait Anger Expression Inventory-2 (STAXI-2) [19]; type of aggression, measured with the Reactive-Proactive Questionnaire (RPQ) [20]; and hostile attribution bias, as measured with the Hostile Interpretation Bias Task (HIBT) [21].

#### 2.5.3. Other Measures

Other measures included: Sociodemographic information, the Child Trauma Questionnaire-Short Form (CTQ-SF) [22]; the Igroup Presence Questionnaire (IPQ) [23], and a short interview at follow-up about experiences with VRAPT was done by a research assistant. The aim of the interview was to collect user experiences and to provide information on the impact, benefits and disadvantages of VRAPT as perceived by the participants.

### 2.6. Statistical Analyses

No prior studies on VR aggression prevention interventions were available. We aimed for a moderate effect size of 0.5 on primary outcomes. Using this effect size with a β-power of 0.80, alpha of 0.05 and an independent two-sided t-test to evaluate the main outcome, 64 subjects were required in each condition, total *N* = 128.

There were missing values on several questionnaires due to unwillingness to complete some questions, not understanding the question, or because they were forgotten. Subscales and total scores were still computed if one missing was present in a scale with less than ten items; for subscales with more than ten items, two missing values were allowed.

Data were analyzed with IBM SPSS Statistics (Version 24.0. Armonk, New York, USA). Significance was accepted at 0.05. To identify any baseline differences between the VRAPT and waiting list group, groups were compared on baseline values of outcome measures, socio-demographic and clinical characteristics with t-tests, chi-squared tests or the non-parametric Mann-Whitney U test. Next, VRAPT completers and VRAPT treatment dropouts were compared on baseline characteristics.

Intention-to-treat analyses were performed on all outcome measures. Outcomes were analyzed with multilevel analysis (MIXED command). Multilevel analyses were performed as these have a hierarchical structure; repeated measures (level 1) are nested within individuals (level 2). Assumptions for multilevel analyses were checked. Multilevel models included fixed effects for time (baseline-post-treatment or baseline-follow-up), group (VRAPT or waiting list) and the interaction time X group, and a random intercept for participant. The covariance structure was set to identity and models were estimated with the maximum likelihood method. For the variables: age, study site, baseline SDAS peak score and highest completed education level there were baseline differences between groups, therefore these variables were included as covariates in all analyses. If Treatment effects were established by the time X group interaction separately for post-treatment (T2) and follow-up (T3) by comparing them separately to pre-treatment (T1). Effect sizes for treatment effects at post-treatment and follow-up were calculated with Cohen’s d, by calculating the effect size for the difference scores T1-T2 as well as T1-T3 between groups [24].

## 3. Results

In total, 128 forensic inpatients were included between 1 March 2017, and 31 December 2018. See Figure 1 for the inclusion flow-chart. Sociodemographic and clinical characteristics are presented in Table 1.

Of the VRAPT group, 67% completed all 16 VRAPT sessions. Thirteen patients (20%) dropped out during the therapy. These drop-out rates are similar to other studies conducted on psychological therapy for aggression in inpatients wards [25,26,27]. Reasons for discontinuation of the VRAPT were: drug use, cardiac arrhythmia, security restrictions, feeling uncomfortable during role-playing, moved to another clinic that was not participating in this study. Eight patients (13%) never started VRAPT after randomization. Patients who completed the VRAPT intervention did not differ significantly from the treatment drop-outs, except for age of first conviction. Treatment drop-outs were on average three years younger (M = 17, SD = 3.60) during their first conviction than the completers (M = 20, SD = 5.65; *t*(57) = 2.02; *p* = 0.05). No serious adverse events were reported and only one adverse event was reported. One participant experienced cardiac arrhythmias and linked this to the physiological measurements in VRAPT. After this incident, he refused to continue VRAPT. Participants felt moderately present in the virtual environments on all three subscales of the IPQ (range 0–6): spatial presence (M = 3.12, SD = 1.1), involvement (M = 2.5, SD = 1.6), and realness (M = 2.18, SD = 1.3).

Means and standard deviations of all outcome measures are presented in Table 2, and test results are presented in Table 3. Concerning primary outcomes, there were no significant changes in staff-rated aggression in both groups. The means of self-reported aggression decreased both in the VRAPT and the control group, though these changes were not significant (T1-T2, F = 1.91(101.04), *p* = 0.17; T1-T3, F = 1.44(100.42), *p* = 0.23), but there was no effect of VRAPT treatment.

From baseline to post-treatment significant treatment effects (time X group interaction) were observed for aggression and hostility (BDHI-D total), direct aggression (subscale BDHI-D), non-planning (subscale BIS-11), anger control—out (subscale STAXI-2), and anger expression index (subscale STAXI-2). In all of these subscales/ total scores, the VRAPT group improved more than the waiting list group. However, these improvements were not maintained at three-month follow-up. No significant treatment effects were found for primary or secondary outcome measures at three-month follow-up. According to the interviews at follow-up, most participants valued VRAPT as addition to their current treatment. Half of them experienced positive changes in their daily life (Table 4).

## 4. Discussion

The present study investigated the effect of a novel virtual reality aggression prevention therapy (VRAPT) in forensic psychiatric inpatients. We engaged and treated a large sample of forensic psychiatric inpatients with aggressive behavior problems. No significant improvements were found after VRAPT compared to waiting list on the primary outcomes: staff-rated aggressive behavior and self-reported aggression. With regard to secondary outcomes, self-reported aggression, anger, hostility and impulsivity decreased in both groups over time. We found positive treatment effects of VRAPT on self-reported direct aggression and hostility, anger control skills, anger expression index and non-planning impulsiveness (i.e., self-control and cognitive complexity). These effects were not maintained at 3-month follow-up.

The findings of this study are in line with the modest results of previous aggression treatment studies in forensic populations [5,6]. Recently, in the Swedish Prison and Probation Services, the largest controlled effectiveness study of ART on recidivism among convicted adult offenders was performed. They compared 1124 offenders who began ART with 3372 matched comparisons offenders who did not receive ART (2003–2009) [28]. Intention-to-treat analyses suggested no advantage for ART in reducing recidivism, although subgroup analyses with ART completers only suggested small reductions in general—not violent—reoffending.

Thus, previous literature indicates that aggressive behavior of forensic patients is not easily changed. Recommendations for improvement of aggression treatments included use of other perspectives than cognitive behavioral therapy, such as individualized training of new skills for real-life risk situations, focusing on destabilizers (i.e., impair decision making), and role-playing as a key component of treatment [29]. Furthermore, it was highlighted that studies that used role-playing were more effective in reducing (violent) re-offending than those that did not [29]. Although our intervention incorporated these recommendations, the results of our study revealed that VRAPT was also not effective in reducing aggressive behavior based on staff rating and self-report of forensic psychiatric inpatients. There are several possible explanations for these results.

First, the content of VRAPT was based on the SIP model [9,30], which is often used as an explanatory model for reactive aggression in children and adolescents, and is also a part of other aggression treatments such as ART [6]. However, this model has some limitations. Early experiences (e.g., childhood trauma) and emotional states are not explicitly represented in the steps of the SIP model, whereas they are quite relevant for aggressive behavior. For instance, childhood trauma (such as emotional abuse or witnessing violence) is highly prevalent among forensic inpatients and has been associated with aggressive behavior in adulthood [31]. Therefore childhood trauma may well be an important determinant of aggression in prisoners [32]. Furthermore, childhood trauma was associated with increased social stress reactivity in a previous VR study [33]. Thus, exploration of impact of childhood trauma on emotions and behavior in social interactions might be a valuable addition to VRAPT.

Second, the VRAPT approach was transdiagnostic. Whereas this design has important advantages in forensic settings, the considerable comorbidity of psychiatric disorders of patients in forensic psychiatry, may have contributed to the lack of significant treatment effects. For instance, appraisals and attributions to other’s behaviors are also dependent on diagnosis. In psychosis, the tendency of patients to perceive others as hostile is linked to paranoid ideation [34]. However, in other disorders, these attributions of intent may be affected by other cognitive biases or psychological mechanisms, which may have been targeted to a lesser degree in VRAPT. Thus, in the current study, the inclusion of patients with different psychiatric disorders may have reduced the overall effect of the VRAPT.

Third, generalization of VRAPT experiences to daily life may have been less than optimal. Participants did not receive homework assignments and were not explicitly encouraged to practice new skills and behavior on the wards between the VRAPT sessions. Furthermore, there was a discrepancy between the VR environments and the patients’ daily life. Some situations, e.g., being in a bar or supermarket, did not directly relate to their life in the highly secured treatment ward. Due to the study design, VRAPT was used as a separate treatment module, and this may have hampered the integration of VRAPT in regular care and treatment.

Fourth, VR might not be suitable for treatment of aggression of forensic psychiatric patients. However, this is not very likely, as the motivation of forensic psychiatric inpatients to participate in VRAPT was high, as evidenced by the high inclusion rate. In addition, most patients and therapists were positive about VRAPT, and during the follow-up interviews, many participants were able to recall what they had learned (e.g., more insight into their triggers; awareness of their physiological arousal). Moreover, during sessions, therapists observed that the VR role-plays clearly elicited emotions and behavior, indicating that participants were able to practice and were immersed in the VR environments.

Fifth, the outcome measures may have been suboptimal. The primary goal of VRAPT was to reduce aggression by improving social information processing mechanisms and training participants to react adequately in provocative and challenging situations. Our findings are similar to the findings from a meta-analysis that showed no significant treatment effect of aggression therapy on institutional behavior [7]. In the current study, behavior and skills in social situations were not explicitly measured, and no validated questionnaires were available to measure the different steps of the SIP model. Furthermore, the use of questionnaires demands a certain level of cognitive skills and insight, which not all participants may have had. In addition, while the SDAS is a behavioral observation scale, it was measured on the wards. Some patients were already working outside the clinic, and some incidents may have remained out of the sight of staff. Further, the SDAS intends to measure subtle forms of aggression, such as irritation, not cooperating with staff and negativism. Staff members in forensic psychiatry are often confronted with more violent forms of aggression and therefore may overlook, or not notice, more subtle forms of aggression.

### 4.1. Strengths and Limitations

A strength of this study was that we successfully conducted a relatively large, rigorous randomized clinical trial of aggression treatment in forensic inpatients, which has hardly been done before [5]. We achieved to include 128 inpatients of four forensic psychiatric centers within 1.5 years. This high inclusion rate seems to reflect a high willingness to engage in VR therapy, which is important as forensic psychiatric inpatients are often hard to motivate. Although drop-out rates are comparable to other studies, most reasons for drop-out were not related to the VR. Second, to the best of our knowledge, this study was the first study to investigate a VR-based treatment in forensic psychiatry. A recent systematic review concluded from pioneering studies that VR is a promising method in forensic psychiatry, but did not find any published VR intervention study [35]. The current study may therefore break new grounds in this field of research.

This study also had several limitations. Self-report measurements may have been too difficult for some participants, as some sentences and statements within the self-report questionnaires required a relatively high language level. Another limitation was a possible selection bias, as the most aggressive inpatients were not approached to participate, because the treatment supervisor did not consent to participation because of possible risks. Participants who dropped-out of VRAPT also had higher average SDAS scores and were on average 3 years younger at their first conviction than the group that completed treatment. A considerable part of the drop-outs had relatively severe aggression problems, thus the training may have been too intensive or confronting for them. However, most reported reasons for drop-out were attributable to treatment motivation problems in general, such as aggression or drug incidents on the wards. In addition, human aggression is complex behavior. Effective therapy for forensic patients with chronic and severe psychiatric and behavioral problems requires thorough and repeated training of new behavior, integrating of several factors, such as the social environment, individual factors and current emotions. It is questionable whether 16 VRAPT sessions were sufficient to achieve this, given an average treatment period of eight years TBS-treatment in a FPC [7]. In addition, within this highly secured setting, it was not possible to keep research assistants blind about the condition of the participants. Research assistants were employees of the institutions and had to have access to the daily reports of the inpatients because of clinical and security reasons. Further, many participants could not be prevented to talk about their VR experiences during the measurements. In the current study, collection of pharmacological data (i.e., medication) was hampered due to limited access into medical files. For future studies, it would be relevant to collect this data and assess the possible influence of medication on the treatment of aggression. Also, in the current study we did not have information on eligibility assessment. Treatment supervisors wanted to screen their patients on eligibility and willingness to participate, before they were visited by the independent research assistant. It was not feasible to collect data on how many patients they had screened. Finally, for secondary outcomes the *p*-value was not corrected for multiple testing this could have resulted in unjustifiably finding an effect.

### 4.2. Implications and Future Research

A key implication of the current study is that it is difficult to establish treatment effects and change aggressive behavior of forensic inpatients. Many forensic inpatients reside in prisons and forensic psychiatric care for many years, and have persistent psychiatric, behavioral and motivational problems. More comprehensive theoretical models and more clinical research are needed to improve efficacy of aggression therapy as part of a comprehensive treatment program for forensic psychiatric inpatients. As participants and therapists repeatedly reported that VRAPT was a relevant addition to patients’ treatment programs and that they enjoyed practicing new behavior in VR, further research of VR aggression treatment is warranted. Effectiveness of VRAPT may be improved by adding and repeating sessions, creating virtual scenarios closer to day-to-day experiences of inpatients, integrating VR with other components of the treatment program and extending biofeedback functionalities.

In future studies, implicit aggression measurements and role-play measures to evaluate social information processing or new behavior in (provocative) social situations may be added to the self-report questionnaires and behavioral observations. One needs to be aware of the highly restricted environment in which the patients are supported by staff to inhibit anger and avoid conflicts. They can present themselves in a socially desirable way, or come across as detached, defiant or uncooperative, which influences staff observations [36]. Therefore, measuring aggressive ideations in a more implicit or experimental way may provide more insight in aggression of forensic psychiatric inpatients. Furthermore, when VRAPT is applied in a sample of participants who are already working or residing outside an FPC, it may be worthwhile to consider observed-rated measures including perceptions of relatives and friends, on top of staff-observations. This could provide a more meaningful reflection of change.

Finally, VR aggression treatment should be investigated in other populations with aggressive behavior problems, such as prisoners in a regular prison ward. VRAPT could also be tested in forensic and non-forensic outpatient samples. Forensic patients that are under semi-restricted measures (i.e., can go outside at particular times), or for those who are about to be discharged may benefit more from VRAPT. Outpatients experience more provocative triggers in their daily life than inpatients in a highly restrictive environment. In addition, it is expected that outpatients may have a more intrinsic request for help to reduce aggressive behavior. Moreover, outpatients are not punished when displaying (minor) aggression, so they are probably more open about misbehaviors. Therefore, these populations might benefit of VRAPT, as they have more opportunities to practice and generalize what they have learned in real-life.

## Figures and Tables

**Table 1:** Baseline characteristics.

	VRAPT Group (*n* = 64)		Waiting List (*n* = 64)				
	M (SD) or *n* %		M (SD) or *n* %				*p*
Age, in years	39.4	(10.6)	38.0	(10.0)		t (126) = 0.80	0.43
Dutch origin	47	73%	51	80%		X^2^(1) = 0.70	0.40
Highest completed education level						X^2^(5) = 4.28	0.51
None	4	6%	6	9%			
Lower education	45	70%	36	56%			
Intermediate education	7	11%	7	11%			
Tertiary education	2	3%	2	3%			
Lower special education	5	8%	12	19%			
Missing	1	2%	1	2%			
DSM-5 diagnosis *							
Schizophrenia	12	19%	14	22%			
Schizoaffective disorder	0	0%	2	3%			
Delusional Disorder	0	0%	2	3%			
Psychotic disorder Not Otherwise Specified (NOS)	6	10%	4	6%			
Paranoid personality disorder	0	0%	1	2%			
Antisocial personality disorder	22	35%	26	41%			
Borderline personality disorder	13	21%	3	5%			
Narcissistic personality disorder	4	6%	3	5%			
Dependent personality disorder	1	2%	1	2%			
Personality disorder NOS	25	40%	27	43%			
Autism spectrum disorder	5	8%	12	19%			
Attention-deficit/hyperactivity disorder	11	17%	8	13%			
Pedophilia	4	6%	4	6%			
Other paraphilias	3	5%	2	3%			
First-offender	4	6%	5	8%		X^2^(1) = 0.12	0.73
Age at first conviction, in years	18.9	(5.2)	18.9	(6.6)		*t* (116) = 0.02	0.99
Type of first offense (*n* = 119) *							
(Attempted) homicide	4	6%	3	5%			
Sexual offense	4	6%	1	2%			
Violent offense	26	41%	26	41%			
Property offense	28	44%	29	45%			
Arson	2	3%	3	5%			
Other	11	17%	10	16%			
Age at index offense, in years	31.3	(9.8)	29.5	(9.2)		*t* (126) = 1.10	0.27
Type of index offense *							
(Attempted) homicide	31	49%	23	36%			
Sexual offense	16	25%	20	31%			
Violent offense	37	59%	37	58%			
Property offense	17	27%	18	28%			
Arson	4	6%	3	5%			
Damage of index offense *							
No injury	10	16%	6	9%			
Physical injury	34	53%	40	63%			
Deadly injury	11	17%	8	13%			
Material damage	15	23%	15	23%			
Psychological neglect	5	8%	7	11%			
Missing	5	8%	4	6%			
Childhood trauma (CTQ-SF)	(*N* = 55)		(*N* = 57)				
Childhood trauma total	53.8	(20.8)	53.5		(20.7)	U = 1502	0.95
Emotional Abuse	11.4	(5.6)	11.0		(5.6)	U = 1508.5	0.55
Emotional Neglect	13.0	(5.4)	13.4		(6.1)	U = 1584.5	0.79
Psychical Abuse	11.2	(6.6)	9.9		(6.2)	U = 1376.5	0.32
Psychical Neglect	10.8	(3.4)	11.0		(2.7)	U = 1787.5	0.26
Sexual Abuse	7.9	(5.4)	8.6		(5.8)	U = 1681	0.45

Note. * These variables do not add to a total score as there is overlap between e.g., diagnoses and types of offenses committed by the same person.

**Table 2:** Summary statistics of primary and secondary outcomes.

		VRAPT						Waiting List Control					
		Pre		Post		Follow-up		Pre		Post		Follow-up	
	Instrument	M	*SD*	M	*SD*	M	*SD*	M	*SD*	M	*SD*	M	*SD*
**Staff-rated**		*n* = 64		*n* = 63		*n* = 62		*n* = 64		*n* = 64		*n* = 62	
Peak aggression (primary outcome)	SDAS	6.9	*6.4*	6.9	*6.7*	6.9	*6.7*	6.2	*6.9*	6.6	*7.0*	6.2	*6.6*
General aggression	SDAS	4.0	*4.5*	4.3	*5.0*	4.1	*5.1*	3.6	*5.0*	3.8	*4.7*	3.6	*4.5*
**Aggression**		*n* = 58		*n* = 43		*n* = 42		*n* = 58		*n* = 51		*n* = 50	
Aggression—total (primary outcome)	AVL	81.0	18.0	73.5	19.1	70.8	18.1	83.1	19.1	76.7	20.8	69.7	21.2
Physical aggression	AVL	27.6	8.6	24.0	8.8	22.0	8.5	28.0	8.3	24.2	9.0	21.5	8.7
Verbal aggression	AVL	14.9	3.0	14.1	3.2	13.9	2.9	14.9	2.9	14.3	3.2	13.3	3.6
Anger	AVL	18.1	5.1	16.3	5.0	15.7	5.1	18.3	5.5	17.5	5.6	16.4	5.8
Hostility	AVL	20.6	5.9	19.0	6.1	19.2	6.4	21.9	7.0	20.7	7.4	18.5	7.1
Reactive and proactive aggression—total	RPQ	18.8	9.4	11.5	10.1	10.0	11.4	18.9	9.5	12.5	10.7	11.8	10.7
Proactive aggression	RPQ	7.6	5.6	4.4	5.3	3.7	6.0	7.8	5.4	5.3	6.3	4.8	5.9
Reactive aggression	RPQ	11.3	4.6	7.1	5.6	6.3	5.9	11.2	5.1	7.3	5.2	7.0	5.5
Aggression and hostility—total	BDHI_D	20.0	6.2	17.2	6.8	18.0	7.2	19.3	6.8	19.9	7.4	18.0	7.6
Direct aggression	BDHI_D	10.1	3.9	7.9	4.4	8.4	4.5	9.3	3.9	8.9	4.0	8.5	4.1
Indirect aggression	BDHI_D	8.0	4.2	6.7	4.4	7.1	4.5	8.0	4.4	8.7	5.0	7.2	5.1
Social desirability	BDHI_D	2.0	1.3	2.5	1.4	2.5	1.3	2.0	1.3	2.3	1.4	2.2	1.3
**Anger**													
State anger	STAXI-2	18.1	6.6	17.0	5.0	18.2	7.3	17.8	5.6	17.8	6.3	16.7	3.9
Trait anger	STAXI-2	18.0	5.2	16.4	4.9	16.5	6.0	17.5	5.5	17.2	6.3	15.8	5.6
Anger expression—Out	STAXI-2	17.0	4.6	15.5	3.8	15.7	4.4	16.0	4.6	16.0	4.5	15.7	4.7
Anger expression—In	STAXI-2	17.1	3.6	17.0	4.3	17.1	4.1	16.9	3.7	16.8	3.6	15.8	3.9
Anger control—Out	STAXI-2	21.2	5.1	23.1	5.5	22.8	5.3	21.0	5.3	20.9	5.0	21.5	5.2
Anger control—In	STAXI-2	22.0	4.9	23.6	5.5	23.9	5.5	22.5	5.7	22.3	5.2	22.6	5.7
Anger expression index	STAXI-2	38.6	12.8	33.7	13.8	34.4	15.2	37.4	13.9	37.6	12.7	35.4	13.6
Anger	NAS-PI	86.9	15.8	80.9	16.1	80.1	17.8	86.9	15.9	84.0	16.7	79.8	17.7
**Other**													
Provocation	NAS-PI	51.4	11.8	48.3	14.0	50.0	15.9	51.0	13.2	49.7	14.0	48.2	13.6
Impulsivity—total	BIS-11	63.5	11.5	59.1	11.2	59.9	11.8	62.9	12.1	62.9	10.7	59.5	12.6
Motor	BIS-11	22.8	4.3	20.9	3.8	21.2	4.3	22.1	4.8	21.6	4.5	20.4	5.2
Cognition	BIS-11	15.7	3.6	14.9	4.1	15.1	3.6	15.9	4.4	16.1	3.8	15.4	4.4
Non-planning	BIS-11	25.0	5.6	23.3	5.3	23.6	5.3	24.8	5.3	25.1	5.1	23.9	5.2
Hostile interpretation bias	HIBT	133.6	76.7	123.7	63.9	131.9	70.8	143.6	75.1	145.0	75.7	132.9	69.6

**Table 3:** Estimates of treatment effects from multilevel regression models.

				Pre-Post							Pre-Follow-up				
		*b*	*t*	df	*p*	95% CI		Effect size	*b*	*t*	df	p	95% CI		Effect size
	Instrument														
**Staff-rated**															
Peak aggression (primary outcome)	SDAS	0.15	0.27	127.3	0.79	−0.98	1.29	0.05	−0.01	−0.02	125.1	0.98	−0.60	0.59	0
General aggression	SDAS	−0.08	−0.17	127.5	0.87	−0.97	0.82	0.03	−0.08	−0.30	125.9	0.76	−0.58	0.43	0.05
**Aggression**															
Aggression—total (primary outcome)	AVL	0.18	0.06	99.8	0.95	−5.63	5.99	0.02	−1.70	−1.10	98.9	0.27	−4.77	1.37	0.19
Physical aggression	AVL	−0.77	−0.55	103.1	0.58	−3.55	2.01	0.11	−0.63	−0.80	104.0	0.42	−2.18	0.92	0.15
Verbal aggression	AVL	0.05	0.10	101.7	0.92	−0.99	1.10	0.01	−0.35	−1.23	102.2	0.22	−0.92	0.22	0.26
Anger	AVL	0.52	0.67	100.8	0.51	−1.03	2.08	0.12	0.13	0.34	98.9	0.73	−0.63	0.89	0.07
Hostility	AVL	0.50	0.42	99.6	0.67	−1.85	2.85	0.12	−0.86	−1.57	97.7	0.12	−1.94	0.23	0.28
Reactive and proactive aggression—total	RPQ	−0.23	−0.12	102.7	0.90	−3.96	3.50	0.37	0.40	0.42	102.2	0.68	−1.49	2.28	0.05
Proactive aggression	RPQ	−0.12	−0.12	100.3	0.91	−2.15	1.92	0.09	0.14	0.28	99.9	0.78	−0.85	1.14	0
Reactive aggression	RPQ	−0.08	−0.08	105.3	0.93	−2.02	1.86	0.03	0.30	0.59	104.5	0.56	−0.70	1.30	0.12
Aggression and hostility—total	BDHI_D	2.74	2.38	99.8	0.02 *	0.45	5.02	0.44	0.12	0.22	96.2	0.83	−0.97	1.20	0.02
Direct aggression	BDHI_D	1.17	2.05	99.0	0.04 *	0.04	2.31	0.38	0.30	1.04	97.5	0.30	−0.27	0.87	0.18
Indirect aggression	BDHI_D	1.66	1.94	102.5	0.06	−0.04	3.36	0.32	−0.08	−0.21	96.2	0.83	−0.82	0.66	0.08
Social desirability	BDHI_D	−0.09	−0.34	102.8	0.74	−0.61	0.43	0	−0.11	−0.83	99.5	0.41	−0.36	0.15	0.12
**Anger**															
State anger	STAXI-2	1.13	0.81	98.0	0.42	−1.64	3.90	0.16	−0.59	−0.78	104.1	0.44	−2.09	0.91	0.10
Trait anger	STAXI-2	0.99	1.26	101.2	0.21	−0.56	2.54	0.25	−0.26	−0.63	99.5	0.53	−1.09	0.56	0.14
Anger expression—Out	STAXI-2	1.32	1.76	101.5	0.08	−0.17	2.81	0.33	0.36	0.95	98.3	0.34	−0.39	1.10	0.18
Anger expression—In	STAXI-2	−0.11	−0.17	101.8	0.86	−1.37	1.15	0.03	−0.54	−1.54	100.5	0.13	−1.23	0.15	0.28
Anger control—Out	STAXI-2	−1.76	−2.40	96.7	0.02 *	−3.22	−0.30	0.48	−0.40	−0.91	94.8	0.36	−1.26	0.47	0.16
Anger control—In	STAXI-2	−1.38	−1.46	102.8	0.15	−3.26	0.50	0.25	−0.67	−1.40	100.3	0.16	−1.61	0.28	0.23
Anger expression index	STAXI-2	4.41	2.21	98.1	0.03 *	0.45	8.36	0.46	0.84	0.80	94.4	0.42	−1.23	2.90	0.17
Anger	NAS-PI	1.21	0.53	98.9	0.60	−3.33	5.75	0.07	−1.11	−0.95	97.1	0.34	−3.44	1.21	0.23
**Other**															
Provocation	NAS-PI	1.85	1.06	98.3	0.29	−1.60	5.31	0.23	−0.88	−0.99	96.1	0.33	−2.65	0.89	0.20
Impulsivity—total	BIS-11	3.43	1.77	100.4	0.08	−0.41	7.27	0.30	−0.23	−0.26	97.3	0.80	−2.02	1.55	0.08
Motor	BIS-11	1.11	1.34	99.1	0.18	−0.53	2.76	0.21	−0.20	−0.49	98.1	0.63	−1.03	0.62	0.15
Cognition	BIS-11	0.73	0.96	103.0	0.34	−0.77	2.23	0.14	0.02	0.05	99.3	0.96	−0.66	0.69	0.01
Non-planning	BIS-11	1.59	2.07	99.3	0.04 *	0.06	3.12	0.39	0.08	0.20	97.6	0.84	−0.68	0.83	0.03
Hostile interpretation bias	HIBT	10.93	0.93	76.3	0.36	−12.57	34.43	0.18	−5.99	−0.88	73.2	0.38	−19.62	7.64	0.27

Note. * *p* < 0.05; the interaction effects of time by treatment condition are shown. Effect sizes for the treatment effect were calculated with Cohen’s *d* on the difference scores T1-T2 and T1-T3 between the groups. *b* = beta coefficient.

**Table 4:** Answers to interview questions for participants of the VRAPT condition who completed the therapy at follow-up (*N* = 38).

Question	Answers	*n* (%)
Did you work on your learning goals?	Yes	26 (68.4%)
	No, or not really	7 (18.4%)
	Don’t know	5 (13.2%)
Do you notice a difference in your daily life?	Yes	19 (50%)
	A little	3 (7.9%)
	No, or not really	16 (42.1%)
Is this therapy an addition to other therapies?	Yes	29 (76.3%)
	No, or not really	4 (10.5%)
	Not for me personally, but for other patients	5 (13.2%)

Note: *n*(%) refers to the number and percentage of participants who provided a certain answer. Data of 4 participants was missing at follow-up.
